# Case Report: Hemophagocytic lymphohistiocytosis in an infant associated with cytomegalovirus infection and X-linked primary immunodeficiency

**DOI:** 10.3389/fimmu.2026.1940119

**Published:** 2026-09-22

**Authors:** Xavier García-Leon, Manuel Gómez del Moral, Daniela Ramirez-Velasategui, Amy Mariuxi Illescas-Romero, Antonio Ramón Gómez-García, Santiago J. Ballaz

**Affiliations:** 1 Departamento de Biología Celular e Histología, Facultad de Medicina, Universidad Complutense de Madrid, Madrid, Spain; 2 Dr. Juan Tanca Marengo National Oncological Hospital- Sociedad de Lucha Contra el Cancer del Ecuador (SOLCA) Guayaquil, Samborondón, Ecuador; 3 Centro de Salud Lojas-Daule, Daule, Ecuador; 4 Centro de Salud Yatubi-Bolivar, Bolivar, Ecuador; 5 Escuela de Posgrado en Salud, Universidad Espíritu Santo, Samborondón, Samborondón, Ecuador; 6 Centro de Investigación Biomédica (CENBIO), Facultad de Ciencias de la Salud Eugenio Espejo, Universidad UTE, Quito, Ecuador

**Keywords:** cytomegalovirus, hemophagocytic lymphohistiocytosis, primary immunodeficiency, XIAP/BIRC4, X-linked lymphoproliferative syndrome

## Abstract

**Background:**

Hemophagocytic lymphohistiocytosis (HLH) is a life-threatening hyperinflammatory disorder often driven by primary immunodeficiencies like X-linked lymphoproliferative syndrome (XLP) in infants.

**Case description:**

We report a 2-month-old male with primary immunodeficiency who developed HLH triggered by cytomegalovirus (CMV), confirmed via bone marrow PCR and hemophagocytosis findings. Initial immunophenotyping during acute hyperinflammation showed marked secondary disruptions across lymphocyte subpopulations, including severe lymphopenia in CD4+ T cells and pre-germinal center B cells. Genetic sequencing identified a pathogenic hemizygous variant in the *XIAP/BIRC4* gene, confirming X-linked lymphoproliferative syndrome type 2 (XLP-2). The patient was managed with the HLH-2004 protocol, valganciclovir, immunoglobulins, and transfusions, complicated by severe pancytopenia.

**Conclusion:**

This case demonstrates an atypical XLP-2 presentation where CMV, rather than Epstein-Barr virus, triggered HLH. Following stabilization and viral clearance, the patient successfully underwent allogeneic hematopoietic stem cell transplantation (HSCT) from a matched family donor. On day +30 post-HSCT, molecular chimerism confirmed 97.87% donor engraftment. On day +39 post-HSCT, the infant is clinically stable, actively recovering at last assessment from grade II cutaneous graft-versus-host disease (GvHD) under immunosuppression, with low-level CMV viral reactivation (951 copies/mL) managed under close surveillance. At the comprehensive 9-month follow-up from initial presentation, the patient maintained overall systemic stability without evidence of disease relapse or secondary organ dysfunction, highlighting the necessity of long-term monitoring in post-transplant cGvHD management, and proving the life-saving role of early genetic diagnosis and timely HSCT.

## 1 Introduction

Hemophagocytic lymphohistiocytosis is a significant systemic inflammatory disorder characterized by persistent immunological deterioration (1). This dysregulation promotes the expansion of activated T lymphocytes, macrophage activation, sustained production of interferon gamma (IFN-γ), interleukins, and other inflammatory mediators, with progressive tissue damage, cytopenias, hyperferritinemia, hypofibrinogenemia, and multi-organ involvement (1, 2). HLH is classified as primary (familial), caused by genetic abnormalities that decrease the cytotoxic capacity of lymphocytes, and secondary (due to infections, neoplasms, or autoimmune diseases) (2–4).

In pediatric age, especially in infants, the identification of HLH requires ruling out innate immune errors. Among them, X-linked inhibitor of apoptosis (XIAP) deficiency, caused by pathogenic variants in the Baculoviral IAP Repeat-Containing protein 4 (*BIRC4*) gene, is currently considered a form of X-linked primary (XLP) immunodeficiency (i.e., XIAP/BIRC4) with a predisposition to recurrent episodes of HLH, inflammatory bowel disease, splenomegaly, hypogammaglobulinemia, and susceptibility to severe viral infections (5–7). Unlike the classic forms of familial HLH due to defects in *PRF1*, *UNC13D*, *STX11*, or *STXBP2* genes, the XIAP/BIRC4 protein deficiency is not limited to a cytotoxicity alteration; it also compromises apoptosis pathways, inflammatory signaling, and regulation of the inflammatory machinery (3, 8, 9).

X-linked lymphoproliferative syndrome (XLP) is an unusual primary immunodeficiency that makes affected male patients susceptible to severe HLH, particularly after viral infections. Although Epstein-Barr virus (EBV) is the most frequently described viral trigger in XLP, particularly in SAP deficiency (5, 10, 11), the cytomegalovirus (CMV) infection can also act as a persistent antigenic stimulus capable of precipitating HLH, especially in patients with underlying primary immunodeficiency (12, 13). The identification of CMV as a trigger in an infant with *XIAP* deficiency is clinically relevant due to its low relative frequency, the need to differentiate it from EBV, and its therapeutic implications, including antiviral treatment, immunomodulation, and early evaluation for allogeneic hematopoietic stem cell transplantation (6, 14).

In this context, we present the case of a male infant diagnosed with HLH associated with primary immunodeficiency with immunophenotypic alterations by flow cytometry and genetic confirmation of XIAP deficiency, highlighting the value of an orderly diagnostic sequence: admission, treatment, relapse, and genetic diagnosis, for which the potentially curative treatment is allogeneic hematopoietic stem cell transplantation (14, 15).

## 2 Case report

### 2.1 Clinical course and complementary studies

Male patient referred from the Hospital Dr. Juan Tanca Marengo-Solca (Guayaquil, Ecuador), with a history of initial admission at 2 months of age for hyperinflammatory syndrome compatible with HLH (**Figure 1**). The condition was associated with a CMV infection documented by PCR in bone marrow, with 1089 x 10^3^ viral copies/mL of plasma. Upon initial evaluation, the patient fulfilled 6 out of the 8 diagnostic criteria established by the HLH-2004 guidelines (**Table 1**) including fever (documented during presentation history), splenomegaly confirmed via abdominal ultrasound and CT, bicytopenia involving two cell lineages, hypofibrinogenemia, hyperferritinemia, and bone marrow aspiration demonstrating profound hemophagocytosis. The remaining criteria (hypertriglyceridemia and low/absent NK cell activity) were either unmeasured or corroborated by functional flow cytometry findings. Markers of severe inflammation and tissue damage were also evident: leukocytes 5230/µL, LDH 3409 UI/L, AST 549 U/L, ALT 1203 U/L, and D-dimer 7.4 µg/mL. The serology showed positive IgG for CMV and negative IgM, a pattern compatible with active infection or reactivation in the described clinical and molecular context.

**Figure 1:**
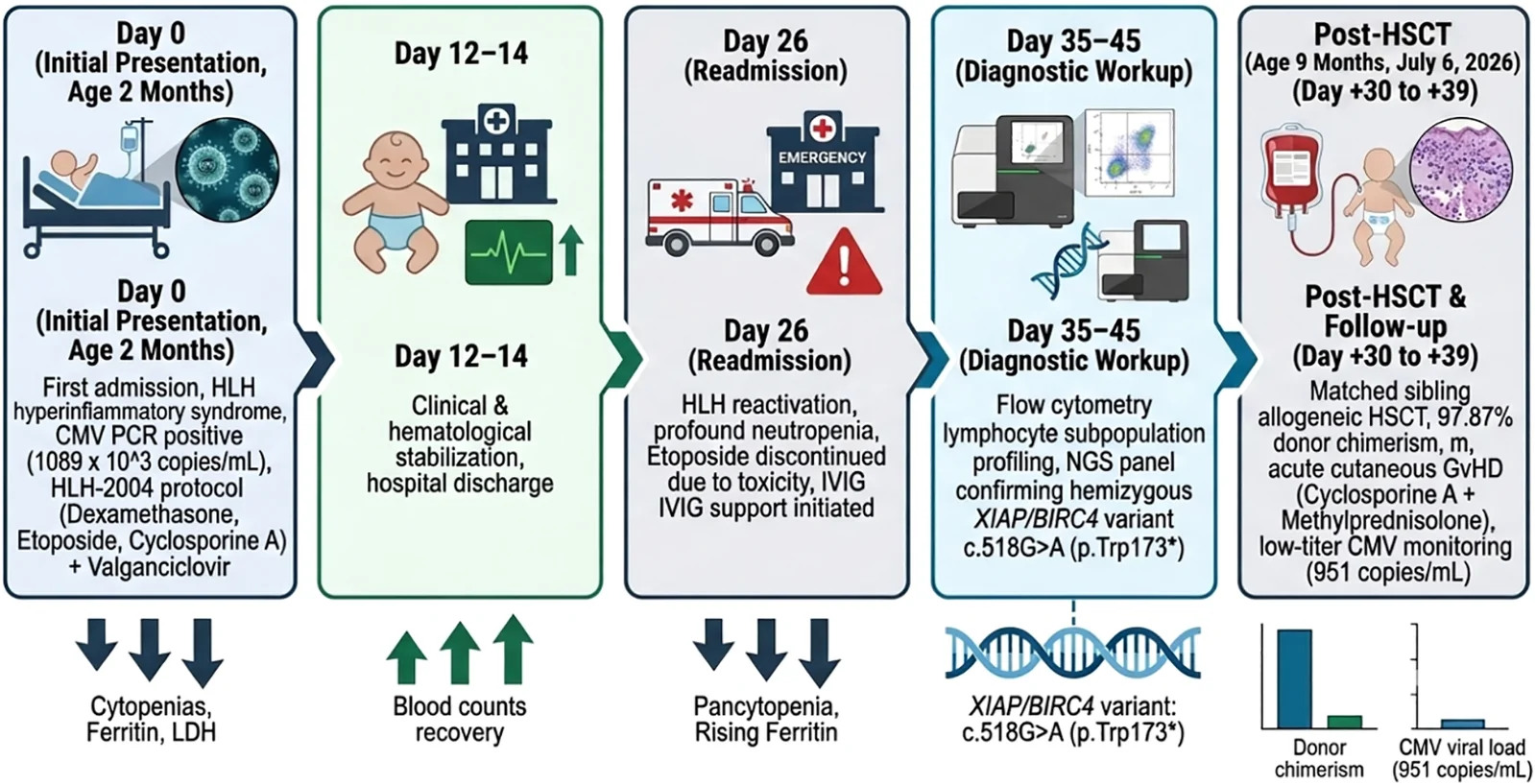
Chronological clinical timeline of the patient, outlining key milestones from initial admission, acute management of CMV-triggered HLH, relapses, immunophenotypic and genetic confirmation of XLP-2, up to day +39 post-HSCT (allogeneic hematopoietic stem cell transplantation). The infant had HSCT at the age of 9 months.

**Table 1:** Fulfillment of HLH-2004 diagnostic criteria.

Criterion	Status
Fever	Present
Splenomegaly	Present
Cytopenias (≥2 lineages)	Present (Hb 6.6 g/dL, Plt 25,000/µL)
Hypertriglyceridemia/Hypofibrinogenemia	Hypofibrinogenemia (114 mg/dL)
Hemophagocytosis in bone marrow	Present (+++)
Low/Absent NK cell activity	Consistent with clinical picture
Hyperferritinemia (>500 ng/mL)	Present (18,150 ng/mL)
Elevated sCD25 (soluble IL-2 receptor)	Not measured

To rule out other viral triggers classically associated with primary immunodeficiencies and secondary HLH, a comprehensive infectious panel was performed (**Table 2**). Epstein-Barr virus (EBV) viral load via quantitative PCR was undetectable, and EBV viral capsid antigen (VCA) IgM and IgG serologies showed no acute activity. Additional screening for human immunodeficiency virus (HIV), toxoplasmosis, rubella, herpes simplex virus (HSV types 1 and 2), and hepatitis B and C yielded negative results, confirming that cytomegalovirus (CMV) was the exclusive active viral pathogen driving the hyperinflammatory cascade.

**Table 2:** Comprehensive microbiological screening panel.

Pathogen	Method	Result
Cytomegalovirus (CMV)	Plasma/BM PCR	Positive (1089 × 10³ copies/mL)
Epstein-Barr virus (EBV)	PCR & VCA (IgM/IgG)	Negative
HIV	Serology	Negative
Toxoplasmosis	Serology	Negative
Rubella	Serology	Negative
Herpes Simplex (HSV 1 & 2)	PCR	Negative
Hepatitis B & C	Serology	Negative

Imaging studies demonstrated systemic involvement. The simple cranial CT scan showed subependymal bleeding without cerebral edema or ventricular dilation. The abdominal ultrasound reported suspected chronic liver disease with inflammatory changes, marked splenomegaly, prominent pancreas, and bilateral hypoxic-inflammatory renal changes. The chest CT showed bilateral interstitial-alveolar opacities predominantly in the parahilar and basal regions, without masses. The abdominal CT scan showed hepatomegaly affecting the left lobe and homogeneous splenomegaly, without pleural or pericardial effusion. The myelogram demonstrated hypercellular bone marrow with marked hemophagocytosis (+++), non-platelet-producing megakaryocytes, and myeloid maturation abnormalities. Overall, the clinical, hematological, biochemical, virological, and medullary findings supported the diagnosis of HLH associated with CMV.

To evaluate the possibility of congenital versus postnatal cytomegalovirus (CMV) acquisition, retrospective neonatal evaluations were reviewed. Although maternal CMV infection during pregnancy is frequently asymptomatic and does not preclude vertical transmission, maternal gestational history was clinically unremarkable for acute infectious symptoms. Retrospective PCR testing of the newborn dried blood spot (Guthrie card) returned a qualitative positive result, confirming congenital cytomegalovirus transmission. Supplementary viral testing during admission confirmed positive saliva CMV PCR, while the infant was exclusively formula-fed, ruling out late post-natal acquisition via breast milk. This congenital infection accounted for the multi-organ involvement including subependymal bleeding, hepatosplenomegaly, and interstitial pneumonitis. Serial quantitative plasma CMV PCR tracking demonstrated a steady decline from 1089 × 10^3^ copies/mL down to undetectable levels following the initial 4-week course of with intravenous ganciclovir (5 mg/kg every 12 hours, subsequently transitioned to oral valganciclovir at 16 mg/kg every 12 hours) combined with targeted HLH therapy. [Note: While standard symptomatic congenital CMV is typically managed with a 6-month course of oral/IV valganciclovir, antiviral therapy in this infant was coordinated alongside cytotoxic and immunomodulatory regimens, with subsequent maintenance and transplant preparation prioritized due to the underlying XLP-2 diagnosis].

### 2.2 Initial treatment

The patient began treatment according to the HLH-2004 protocol (2, 12), receiving dexamethasone, etoposide, and cyclosporine A, along with supportive measures. During hospitalization, he received red blood cell concentrates transfusion at 10 mL/kg, serial platelet transfusions, broad-spectrum antibiotic therapy, intravenous ganciclovir (or oral valganciclovir) for four weeks, and weekly intravenous immunoglobulin as immunomodulatory support. The decision to combine HLH-targeted therapy and antiviral treatment was based on the coexistence of severe hyperinflammation and molecular evidence of CMV (11).

### 2.3 Evolution

Twelve days after discharge, the patient was readmitted due to clinical and hematological reactivation of HLH. During his second admission, persistent bicytopenia associated with profound neutropenia was observed, leading to the discontinuation of etoposide due to toxicity manifesting as myelosuppression (2, 16). Following the suspension of etoposide due to toxicity and to counteract the ongoing hyperinflammation, intravenous immunoglobulin was initiated weekly for four weeks as standard treatment. Due to his immunodeficiency and evidence of signs of hyperinflammation, it was decided to initiate immunoglobulin treatment (2, 16). In a follow-up complete blood count, leukocytosis with neutrophilia was observed, interpreted as a probable concomitant infectious process or a side effect of the colony-stimulating factor, which was also suspended.

### 2.4 Immunophenotypic and genetic diagnosis

#### 2.4.1 Immunophenotypic study

The recurrence of HLH episodes, early onset, male sex, and unfavorable clinical progression motivated the study of primary immunodeficiency. Flow cytometry in peripheral blood demonstrated extensive alterations in lymphoid and myeloid subpopulations (**Table 3**). Compared to healthy controls, a marked reduction in pre-germinal center B cells was observed, with 70 cells/µL (reference value: 455–3585 cells/µL), naive CD4+ T lymphocytes at 652 cells/µL (reference value: 1100–5170 cells/µL), CD4−CD8−/dim TCRγδ+ T lymphocytes at 38 cells/µL (reference value: 45–430 cells/µL), NK cells at 138 cells/µL (reference value: 180–1330 cells/µL), monocytes at 254 cells/µL (reference value: 390–2950 cells/µL), and plasmacytoid dendritic cells at 3 cells/µL (reference value: 9–60 cells/µL). (**Supplementary Figures 1**, **2**) (17, 18).

**Table 3:** Peripheral blood lymphocyte subpopulation immunophenotyping and age-matched EuroFlow reference ranges.

Estimated cellularity in relation to the total number of nucleated cells examined		
Population	Events/ul	Range/ul
Linphocytes	2150	2500-9920
B- cells	88	485-3950
Pre-Germinal Centre	70	455-3585
Post-Germinal right (post-GC CBM)	17	8-405
BCM ‘‘Unswitched¨/Plasmatic cells (CP)	13	3-340
BCM ¨Switched¨/CP	4	4-100
T-cells	1924	1715-7500
CD4+CD8-	1013	1275-5460
Naive CD4+	652	1100-5170
CD4+Central/Transitional Memory	279	150-835
CD4+ Effector Memory	80	0.2-40
Effector TD CD4+	2	0-7
CD8+ CD4-	714	370-2000
Naive CD8+	373	340-1805
CD8+ central/transitional memory	84	25-475
Effector memory CD8+	87	0-135
CD8 + TD Effectors CD27+d	26	0-365
CD8+ TD Effectors CD27-	145	0-220
CD4-CD8-/dim TCRgd+	38	45-430
CD4-CD8-/dim TCRgd-	157	10-70
NK cells	138	180-1330
Eosinophils	82	50-695
Neutrophils	417	775-8800
Monocytes	254	390-2950
Monocytes CD16+	70	5-75
Plasmocytoid dendritic cells	3	9-60
Basophils	18	5-75

Patient values are expressed as absolute counts (events/µL). Reference ranges correspond to the 5^th^–95^th^ percentiles of the healthy reference cohort aged 1–11 months old (n= 23), established using the standardized EuroFlow Primary Immunodeficiency Orientation Tube protocol.^21.^

#### 2.4.2 Genetic diagnosis

These findings pointed toward a combined alteration of cellular and humoral immunity. Using next-generation sequencing (NovaSeq 6000 system, Illumina, Inc., San Diego, CA, USA) according to the manufacturer’s instructions, deletions and duplications were analyzed in a panel of 1415 genes, identifying a hemizygous pathogenic variant in *XIAP/BIRC4* [c.518G>A (p.Trp173*)], compatible with X-linked lymphoproliferative syndrome type 2 (XLP-2, **Figure 2**). To our knowledge, this specific XIAP variant has not been previously reported in published literature or public genomic databases (e.g., gnomAD, ClinVar). Additionally, a heterozygous pathogenic variant in *TRNT1* [c.748delinsAAGAATGTAAATCAA] was identified, associated with autosomal recessive sideroblastic anemia and retinitis pigmentosa; however, since it was found in heterozygosity and no sideroblasts were observed in the bone marrow, it was considered insufficient to explain the main clinical phenotype.

**Figure 2:**
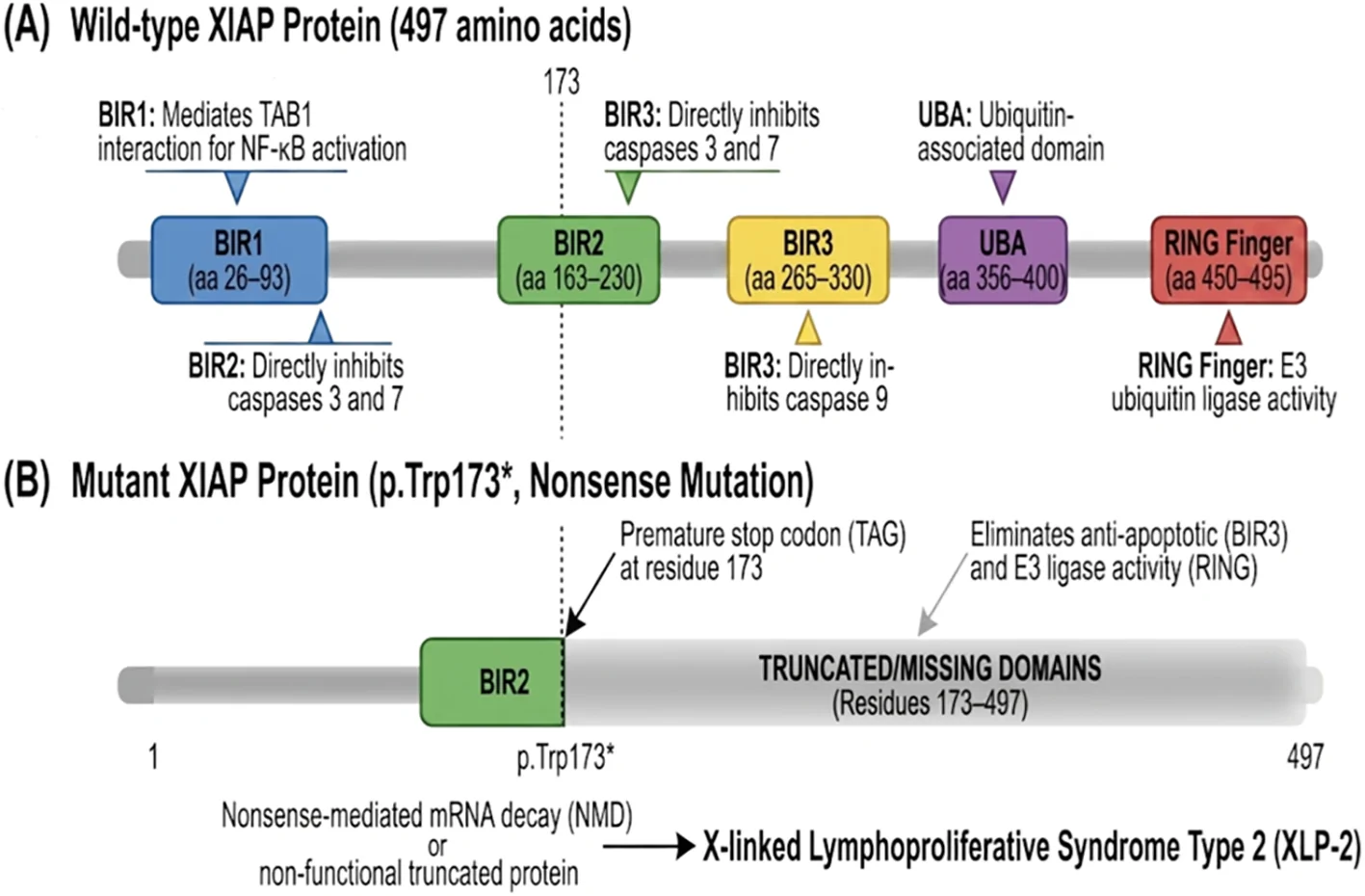
Schematic representation of the human XIAP protein domain structure, highlighting the approximate location of the nonsense mutation p.Trp173* identified in this patient. **(A)** Wild-type XIAP protein (497 amino acids) containing three Baculovirus IAP Repeat domains (BIR1, BIR2, BIR3), a Ubiquitin-Associated domain (UBA), and a C-terminal RING-finger domain. **(B)** The hemizygous nonsense mutation c.518G>A (p.Trp173*) located within the BIR2 domain introduces a premature stop codon at codon 173, resulting in severe truncation (residues 173–497). complete loss of the BIR3, UBA, and RING domains. and protein deficiency characteristic of X-linked lymphoproliferative syndrome type 2 (XLP-2).

Additional variants of uncertain significance are shown in **Table 4**.

**Table 4:** Additional variants of uncertain significance (VUS) and incidental copy number variations (CNVs).

Gene	Variant	Type
ALG12	Gene gain	CNV (duplication)
C8A	c.1450C>T (p.Arg484Cys)	Missense
CA5A	Gene gain	CNV
CD19	c.1469G>A (p.Arg490Gln)	Missense
CTSF	c.215C>T (p.Ala72Val)	Missense
GAN	c.581A>T (p.Tyr194Phe)	Missense
HERC1	c.10964C>A (p.Ser3655Tyr)	Missense
HERC1	c.3715A>G (p.Ser1239Gly)	Missense
HNRNPU	c.1453G>A (p.Val485Ile)	Missense
LRBA	c.4547G>A (p.Arg1516Lys)	Missense
MLC1	Ganancia (copia completa)	CNV
NDUFA12	c.398_400del (p.Lys133del)	Delection in-frame
POLG	c.3445G>A (p.Ala1149Thr)	Missense

To ensure diagnostic transparency and a comprehensive genomic overview, **Table 5** documents co-occurring genetic alterations identified by the 1415-gene next-generation sequencing panel. All identified variants were rigorously evaluated and filtered in accordance with the standard joint consensus recommendations of the American College of Medical Genetics and Genomics and the Association for Molecular Pathology (ACMG/AMP) guidelines, alongside comprehensive genotype-phenotype correlation. Notably, despite the presence of variants in key immune-related loci such as LRBA and CD19, the patient’s acute hyperinflammatory clinical presentation, specialized flow cytometry lymphocyte subpopulation patterns, and bone marrow findings did not align with the disease phenotypes typically driven by pathogenic mutations in these genes. Consequently, under the ACMG/AMP framework, these alterations were classified as benign incidental findings or passenger VUS, validating the hemizygous pathogenic variant in *XIAP/BIRC4* as the definitive and primary pathophysiological driver of XLP-2 and secondary hemophagocytic lymphohistiocytosis (HLH) in this infant.

**Table 5:** Classification and zygosity of detected genetic variants in XIAP and TRNT1.

Gen	Variant	Zygosity	Classification
*XIAP*	c.518G>A (p.Trp173*)	hemizygotic	Pathogenic
*TRNT1*	c.748delinsAAGAATGTAAATCAA	heterozygotic	Pathogenic

Due to the patient’s critical condition and the immediate clinical necessity to define a therapeutic strategy, functional confirmation via intracellular flow cytometry or Western blot for XIAP protein expression in peripheral blood mononuclear cells was not performed. The pathogenicity of the nonsense variant c.518G>A (p.Trp173*) was established based on the genetic evidence of a truncating mutation (**Figure 2**), the critical nature of the acute clinical presentation, and its strong clinical correlation with the XLP-2 phenotype.

### 2.5 Post-transplant outcomes and clinical follow-up

Following initial disease stabilization and viral clearance, the patient proceeded to definitive allogeneic hematopoietic stem cell transplantation (HSCT) using a matched non-carrier sibling donor. On day +30 post-HSCT (evaluated on August 5, 2026), single nucleotide polymorphism (SNP) genomic profiling demonstrated a donor chimerism of 97.87% (and 2.13% recipient), confirming successful myeloid and lymphoid engraftment. On day +39 post-HSCT (August 14, 2026; total active follow-up of 9 months, patient aged almost 1 year), the infant remains hemodynamically stable. His post-transplant course has been complicated by acute cutaneous graft-versus-host disease (GvHD) affecting >40% of the body surface area (presenting as resolving erythematous macules/micro papules without active epidermal sloughing), which was responding favorably to dual immunosuppressive therapy with Cyclosporine A and Methylprednisolone (2 mg/kg/day). Crucially, routine post-transplant molecular surveillance revealed low-titer CMV viral reactivation in peripheral blood (389 copies/mL, rising to 951 copies/mL following a negative PCR). This low-level reactivation underlined the high diagnostic and prognostic value of serial quantitative CMV PCR monitoring in *XIAP*-deficient patients. In this setting, CMV represents both the primary infectious trigger of the initial hyperinflammatory crisis and an ongoing opportunistic threat during post-transplant immune reconstitution and GvHD-induced pharmacologic immunosuppression. Total clinical follow-up spanned 9 months from initial presentation (patient aged almost 1 year old). At 9 months post-transplantation, routine viral surveillance confirmed a negative CMV quantitative PCR result, with no clinical or laboratory evidence of reactivation. The patient continues to show full donor chimerism and complete donor-derived reconstitution.

### 2.6 Ethical considerations and informed consent

This report was conducted in accordance with the Declaration of Helsinki and CARE guidelines. The study conformed to institutional ethical standards of the Hospital Dr. Juan Tanca Marengo-SOLCA (Guayaquil, Ecuador). Due to the patient’s age (2-month-old), written informed consent and pediatric proxy assent were fully obtained, and signed by the parents. Following ICMJE guidelines, confidentiality was strictly maintained.

## 3 Discussion

The main observation was the association between HLH triggered by CMV and associated with XIAP deficiency in an infant, a combination less common than EBV-linked HLH within the spectrum of XLP (7, 10).

In XLP-2, susceptibility to HLH does not solely depend on a classic cytotoxic failure, but rather on a complex alteration of immune homeostasis (14, 15). XIAP is involved in the regulation of apoptosis, the modulation of pattern recognition receptor-dependent inflammatory signals, and the control of inflammasome activation (9, 15). When XIAP is dysfunctional, viral stimuli can generate a disproportionate, persistent, and difficult-to-shut-off immune response (19).

Initial flow cytometry during presentation revealed severe peripheral lymphopenia, including reduced pre-germinal center B cells (70/µL) and naive CD4+ T cells. While XIAP plays a role in lymphocyte survival and apoptosis regulation, this marked suppression during acute presentation was heavily confounded by acute HLH-induced tissue damage, severe cytokine storm, and ongoing CMV replication, precluding its attribution solely to the genetic defect (9, 15). Its deficiency significantly impairs early B-cell development and compromises survival thresholds. Crucially, this cytopenia was further exacerbated by the severe systemic hyperinflammation and the immense cytokine storm (characterized by elevated IFN-γ and TNF levels) that accompanies acute HLH episodes, compounding the initial bone marrow suppression and destruction induced by the active CMV infection itself (3, 12, 20).

In this patient, CMV likely acted as a sustained antigenic stimulus capable of activating T lymphocytes, NK cells, and monocytes/macrophages in a genetically predisposed environment (12). Although EBV is the prototypical viral trigger of XLP, the absence of evidence of EBV and the detection of CMV by PCR in bone marrow support CMV as the main trigger of the episode (10, 12, 13). The relevance of this point lies in the fact that CMV can induce intense immune activation, but in immunocompetent individuals, it rarely progresses to fulminant HLH. The progression to a severe hyperinflammatory phenotype suggests that *XIAP* deficiency reduced the threshold necessary to develop a cytokine storm (10, 15).

From a pathological standpoint, XIAP dysfunction can promote HLH through three complementary pathways. First, the alteration of apoptosis prevents an adequate contraction of activated immune populations, prolonging the inflammatory signal. Second, abnormal inflammasome signaling can amplify the production of mediators such as IL-1β and IL-18, which enhance the activation of T lymphocytes and macrophages (9, 14, 15). Third, the persistence of cellular activation promotes a cytokine storm dominated by IFN-γ, IL-6, TNF, and interleukins dependent on JAK/STAT pathways, with secondary macrophage activation, hemophagocytosis, and tissue damage. Physiologically, this mechanism of antigen persistence and failure to eliminate infected cells is visualized in **Supplementary Figures 3**, **4**. This combination of extreme hyperferritinemia, hypofibrinogenemia, cytopenias, hepatic involvement, and medullary hemophagocytosis is the classic diagnostic hallmark observed in these cases (1, 8).

Flow cytometry was decisive because it provided early evidence of a primary immunodeficiency before genetic confirmation (16, 21). The reduction of pre-germinal center B cells, naive CD4+ T lymphocytes, NK cells, monocytes, and plasmacytoid dendritic cells does not constitute a molecular diagnosis on their own. However, in the context of recurrent HLH, onset in infancy, and male sex, it pointed toward a combined immunological disorder and justified the extended genetic sequencing (20). The confirmation of the hemizygous pathogenic variant in *XIAP/BIRC4* allowed for the planning of allogeneic hematopoietic progenitor cell transplantation (2, 19). The current management of cytokine storm through targeted therapies is essential to stabilize the patient before such a curative therapeutic procedure, as shown in **Supplementary Figure 5** (8, 19).

Following genetic confirmation of XLP-2, targeted anti-cytokine therapies—including the JAK1/2 inhibitor ruxolitinib and the anti-IFN-γ monoclonal antibody emapalumab—were administered as a tailored bridge to allogeneic HSCT, with selection dictated by regional pharmacological availability. Because persistent IFN-γ hypersecretion drives the core immunopathology of *XIAP/BIRC4* deficiency, direct upstream neutralization with emapalumab and downstream signaling blockade with ruxolitinib offered effective control of the hyperinflammatory state while significantly minimizing systemic chemo-toxicity prior to definitive transplantation.

The confirmation of the hemozygous pathogenic variant in *XIAP/BIRC4* changed the interpretation of the clinical picture. The case ceased to be merely HLH secondary to viral infection and came to be considered HLH in the context of X-linked primary immunodeficiency. This distinction has important therapeutic consequences: it allows for anticipating recurrences, selecting a non-carrier family donor, avoiding attributing the entire evolution to isolated infection, and planning for allogeneic hematopoietic progenitor cell transplantation when the clinical condition permits. The identification of a non-carrier, histocompatible sibling reinforces the clinical applicability of this approach. The post-transplant course in *XIAP/BIRC4* deficiency is frequently complicated by opportunistic viral reactivations, particularly CMV, during the early post-engraftment phase before robust T-cell mediated immunity is re-established (14). In our patient, despite successful donor cell engraftment by day +30 post-transplant, CMV viral reactivation recurred on hospital day +39. This highlights the vital importance of continuous quantitative viral PCR surveillance in XLP-2 patients post-HSCT, where prompt re-institution of targeted antiviral therapies (such as oral valganciclovir or intravenous ganciclovir) is essential to prevent secondary graft dysfunction or disease flares during early immune reconstitution.

The heterozygous variant in *TRNT1* should be interpreted with caution. Although *TRNT1* is associated with autosomal recessive sideroblastic anemia, variable immunodeficiency, and multisystemic alterations, the isolated finding in heterozygosity, along with the absence of sideroblasts in the bone marrow, does not sufficiently explain the dominant phenotype of recurrent HLH. Therefore, it is considered an additional genetic finding that should be documented, but it does not displace *XIAP* as the main pathophysiological axis of the case.

The novelty of the case is based on three elements. First, HLH triggered by CMV in *XIAP* deficiency is rare compared to the classic association with EBV (7). Second, flow cytometry allowed for early suspicion of an integrated defect in cellular and humoral immunity, guiding the indication for genetic analysis. Third, molecular confirmation allowed for decisions oriented toward early transplantation, including histocompatibility evaluation and exclusion of carriers within the family. Overall, the case illustrates how the integration of molecular virology, flow cytometry, and genetics can modify the diagnostic and therapeutic trajectory of infants with HLH.

A critical takeaway from this case is the diagnostic trajectory. Because the patient presented with a high-titer CMV viral load and multi-systemic manifestations overlapping with congenital infection, the diagnostic workup rapidly escalated to genetic sequencing. Had the CMV infection not been so prominent, this infant might have initially been managed purely as secondary infection-driven HLH, delaying the critical identification of XLP-2 until later classic manifestations (such as recurrent HLH, colitis, or hypogammaglobulinemia) emerged. This underscores the necessity of maintaining a low threshold for early genetic screening in infants presenting severe viral-triggered hyper inflammatory syndromes.

While the broad peripheral blood lymphocyte subset alterations (such as reduced naive CD4+ T cells, pre-germinal center B cells, and NK cells) initially raised suspicion for an underlying immune defect, these findings must be interpreted with caution. In the acute setting of fulminant HLH compounded by persistent viral replication and intensive immunosuppressive treatments (dexamethasone, etoposide, cyclosporine, and IVIG), severe secondary cytopenias and lymphocyte depletion are common and can heavily confound immunophenotypic profiles (22).

The gold-standard screening approach for suspected X-linked lymphoproliferative syndromes relies on functional assays and protein-specific flow cytometry—specifically evaluating targeted pathway functional testing. Rather than cytotoxicity assays, functional screening for XIAP deficiency evaluates monocyte NOD2 signaling via muramyl dipeptide (L18-MDP) stimulation, which yields blunted TNF responses (23). In our patient, the identification of the nonsense variant c.518G>A(p.Trp173*) dictates a complete absence of functional XIAP protein expression. Because specialized intracellular protein flow cytometry panels were restricted at our local center during the acute crisis, whole exome sequencing (WES) was deployed as the primary diagnostic modality. Given that active hyperinflammation significantly worsens allogeneic hematopoietic stem cell transplantation (HSCT) outcomes in XIAP deficiency, minimizing the diagnostic delay between presentation and genetic confirmation is paramount (23).

A notable limitation of this case report was that, in the acute phase, focus was placed on rapid molecular genetic sequencing to secure a diagnosis. Functional confirmation of XIAP protein absence via Western blot or flow cytometry was deferred due to the critical nature of the patient’s clinical presentation and the immediate need to finalize transplant planning. Another significant limitation was the absence of repeat lymphocyte subset immunophenotyping following the complete clearance of the CMV infection, and resolution of hyperinflammation. Consequently, without post-clearance baseline values, the observed reductions in naive CD4+ T cells and pre-germinal center B cells cannot be definitively attributed to the intrinsic XLP-2 cellular phenotype, as they likely reflect transient secondary suppression induced by active viral replication, extreme cytokine storm, and intensive cytotoxic/immunosuppressive therapy. Therefore, these immunophenotypic alterations must be interpreted with caution and cannot serve as diagnostic or supporting evidence for XIAP deficiency.”

## 4 Conclusions

A case of HLH associated with CMV in an infant with XIAP deficiency confirmed by genetic sequencing is presented. The case is relevant because it documents a less common viral trigger than EBV within the spectrum of XLP-2 and shows how flow cytometry can guide the suspicion of primary immunodeficiency earlier (21). The molecular confirmation of the *XIAP/BIRC4* gene allowed for the reinterpretation of HLH recurrence as an expression of an innate immune error, facilitating the planning of allogeneic hematopoietic progenitor transplantation with the selection of a compatible non-carrier family donor.^2^ The chronological integration of admission, treatment, relapse, and genetic diagnosis strengthens the clinical value of the report and highlights the need to study primary immunodeficiencies in infants with recurrent HLH or triggered by unconventional viral infections (1, 24).

## Data Availability

The original contributions presented in the study are included in the article/**Supplementary Material**. Further inquiries can be directed to the corresponding author.
