# Supplementary material for: Case Report: Hemophagocytic lymphohistiocytosis in an infant associated with cytomegalovirus infection and X-linked primary immunodeficiency
Source: Front Immunol. 2026 Sep 22;17:1940119. doi: 10.3389/fimmu.2026.1940119 (PMC13638413; doi:10.3389/fimmu.2026.1940119)
Supplement: Supplementary file 1 [file Supplementaryfile1.docx]

**Supplementary Figure 1. Peripheral blood B-cell maturation defect in XIAP deficiency (XLP-2): Severe pre-germinal center B-cell depletion and borderline switched memory B-cells**

Flow cytometric analysis of peripheral blood B-cell subsets in healthy control (upper panels) and the patient with XIAP/BIRC4 deficiency (XLP-2) (lower panels). Absolute counts (cells/µL) are shown. Reference intervals correspond to age-matched values published by EuroFlow for the Primary Immunodeficiency Orientation Tube [23,24].

- A severe reduction of pre-germinal center B cells was identified (70 cells/µL; reference interval 455–3585 cells/µL).
- Switched memory B cells/plasma cells (BCM switched/PC) were detected at the lower limit of normal (4 cells/µL; reference interval 4–100 cells/µL).
- These findings indicate impaired early B-cell maturation and defective germinal center differentiation, compatible with the humoral immunodeficiency associated with XIAP/BIRC4 deficiency.

**Supplementary Figure 2. Naïve CD4+ T-cell lymphonia in XIAP deficiency (XLP-2): Marked reduction of naïve CD-4 T-lymphocytes**

Flow cytometric analysis of peripheral blood CD4+ T-cell subsets in a healthy control (upper panels) and a patient with XIAP/BIRC4 deficiency (XLP-2) (lower panels). Absolute counts (cells/µL) are shown. Reference intervals correspond to age-matched values published by EuroFlow for the Primary Immunodeficiency Orientation Tube [21].

- The patient exhibits a marked reduction of naïve CD4+ T lymphocytes: 652 cells/µL (reference interval 1100–5170 cells/µL).
- The reduction is consistent across different gating strategies (CD45RA, CD27, and combined analyses).
- This finding indicates impaired thymic output and/or survival of naïve CD4+ T cells, compatible with the combined immunodeficiency associated with XIAP/BIRC4 deficiency.

**Supplementary Figure 3.** Defective cytotoxic granule exocytosis and persistent immune activation in hemophagocytic lymphohistiocytosis**.**

Effective elimination of infected cells requires coordinated polarization and release of perforin- and granzyme-containing cytolytic granules toward the immunological synapse. Genetic defects affecting cytotoxic pathways result in impaired clearance of antigen-presenting targets, persistent immune stimulation, sustained cytokine production, and progression to HLH. This pathway represents a central pathogenic mechanism shared by primary and secondary forms of HLH. Schematic diagram adapted from Meyer et al [14].
**Supplementary Figure 4.** Mechanistic paradigms of CMV-induced NLRP3 inflammasome hyperactivation, positive cytokine feedback loops, and systemic hyperinflammation driven by hemizygous XIAP/BIRC4 deficiency.

Hemizygous pathogenic mutations in XIAP/BIRC4 disrupt crucial anti-apoptotic, autophagic, and ubiquitination pathways, significantly lowering the activation threshold for systemic inflammation. In the context of an active CMV infection—serving as an unconventional primary trigger—persistent pathogen- and damage-associated molecular pattern (also known as PAMP/DAMP) signaling induces the aberrant and unrestrained assembly of the NLRP3 inflammasome. This hyperactivation accelerates caspase-1-mediated processing of pro-IL-1β and pro-IL-18 into their mature forms, fueling a self-sustaining positive feedback loop that culminates in a severe systemic cytokine storm dominated by IFN-γ, TNF-α, and IL-6. Ultimately, this non-resolving hyperinflammatory cascade drives extensive macrophage overactivation, pathological hemophagocytosis, and multi-organ failure, establishing the definitive clinical phenotype of secondary HLH. Schematic diagram adapted from Dissanayake et al [9].

**Supplementary Figure 5.** Targeted therapeutic interventions and molecular strategies for mitigating the hyperinflammatory cascade and cytokine storm in XIAP deficiency.

This explanatory schema summarizes the targeted therapeutic continuum designed to systematically suppress the hyperinflammatory cascade and safely bridge patients toward immune reconstitution. Frontline cytoreduction and immunosuppression via the HLH-2004 protocol (dexamethasone, etoposide, and cyclosporine A) target aberrant lymphocyte proliferation and initial baseline hypercytokinemia. To control the driving core of the cytokine storm, targeted biological therapies block specific extracellular and receptor-mediated pathways: emapalumab directly neutralizes circulating IFN-γ to arrest pathological macrophage overactivation, while anakinra and tocilizumab antagonize anomalous IL-1β signaling and IL-6 receptor-mediated loops, respectively. Intracellularly, the downstream transcriptional amplification of these synergistic inflammatory signals is intercepted via JAK1/2 inhibition with ruxolitinib. Essential adjunctive care prioritizes targeted antiviral therapy (ganciclovir/valganciclovir) to eradicate the sustained CMV antigenic stimulus, combined with immunomodulatory intravenous immunoglobulins (IVIG) and transfusion support. Ultimately, allogeneic hematopoietic stem cell transplantation (HSCT) represents the definitive, exclusive curative strategy capable of correcting the hemizygous XIAP/BIRC4 defect, restoring physiological cell survival thresholds, and re-establishing long-term immune tolerance. Schematic diagram adapted from Zhou and colleagues [16].
